# Quality of life more than 10 years after radiotherapy for localized prostate cancer—impact of time after treatment and prescription dose

**DOI:** 10.1007/s11136-020-02639-7

**Published:** 2020-10-09

**Authors:** Michael Pinkawa, Amr Gharib, Marsha Schlenter, Ludmila Timm, Michael J. Eble

**Affiliations:** 1 Department of Radiation Oncology, RWTH Aachen University, Pauwelsstrasse 30, 52072 Aachen, Germany; 2 Department of Radiation Oncology, MediClin Robert Janker Klinik, Villenstr. 8, 53129 Bonn, Germany

**Keywords:** Prostate neoplasms, Conformal radiotherapy, Quality of life, Dose–response relationship

## Abstract

**Purpose:**

Analysis of quality of life changes after radiotherapy with focus on the impact of time after treatment and prescription dose.

**Methods:**

Consecutive patients were treated with doses from 70.2/1.8 Gy (n = 206) to 72/1.8–2.0 Gy (n = 176) in a single centre and surveyed using the Expanded Prostate Cancer Index Composite questionnaire.

**Results:**

Urinary and bowel bother scores decreased 1 / 3 / 6 points and 7 / 7 / 9 points on average 1 / 5 / 10 years after RT in comparison to baseline scores. The rate of urinary (need of pads in 8% vs. 15% before vs. 10 years after RT; p = 0.01) and bowel (uncontrolled leakage of stool in 5% vs. 12% before vs. 10 years after RT; p < 0.01) incontinence, as well as rectal bleeding (4% vs. 8% before vs. 10 years after RT; p = 0.05) increased. Sexual function scores decreased (erections sufficient for intercourse in 36% vs. 12% before vs. 10 years after RT; p < 0.01).

A higher dose had a statistically significant impact on urinary bother and stool incontinence, but also tended to decrease urinary continence. Age and comorbidities did not have an influence on score changes, but on baseline urinary function/bother and baseline sexual function.

**Conclusion:**

Apart from an increasing rate of erectile dysfunction, urinary and bowel incontinence rates increased with increasing follow-up period. A higher dose was found to be associated with increased urinary problems and larger stool incontinence rates. Age and comorbidities were found to be relevant for baseline scores, but not for score changes.

## Introduction

External beam radiotherapy (RT) is an established curative treatment for prostate cancer. Several technical advances have been introduced in the last two decades, as image-guided radiotherapy (IGRT) or intensity-modulated radiotherapy (IMRT). Smaller treatment volumes with improved prostate localization and better dose conformity with improved dose delivery allow a dose escalation [1, 2]. Several prospective randomized dose escalation studies, using three-dimensional conformal techniques, have demonstrated increasing biochemical control rates with higher prescription doses. However, higher doses have also been shown to be associated with increased rectal toxicity [3]. Overall survival advantages have been shown in subanalyses of randomized studies [4] or non-randomized studies [5].

Health-related quality of life (QoL) has been increasingly recognized to be an essential part of treatment evaluation, describing treatment-associated effects from the patient`s perspective. Studies reporting QoL after a follow-up of more than 5 years in relation to baseline values are hardly available [6]. As radiotherapy is known to have effects even many years after treatment, long-term effects on QoL are of high practical relevance [7].

The most important new contribution to the literature is the QoL analysis after > 10 years in patients treated within normal clinical routine. In contrast to tumour control, this information is not available in the completed dose escalation studies. Consequently, as treatment techniques change with time, the treatment in these patients corresponds to the standards used > 10 years ago.

Comprehensive QoL evaluation has been introduced early for our patients, so that currently QoL results > 10 years after RT are available. Prescription dose has been increased over the years, so that not only the effects of higher doses on tumour control and survival, but also on QoL have been analysed with a specific focus on the impact of total dose.

As long-term toxicity following radiotherapy is extremely important, especially for patients with a long life expectancy, but poorly investigated, the results of this study are novel and urgently needed.

Dose escalation is always an issue in prostate cancer radiotherapy, as tumour control rates can be increased with higher doses. Understanding the effects of dose escalation on QoL many years after treatment is another novel and practically relevant aim of this study.

## Methods

A group of 382 consecutive patients with localized T1-T3N0M0 prostate cancer was treated in the years 2003–2006 with a three-dimensional conformal four-field box technique with 15 MeV photons and a multileaf collimator in a single institution, corresponding to the routine treatment at this time. The PTV was required to be enclosed by the 90% isodose relative to the reference point with a margin of 1.5 cm in the anterior/lateral and 1 cm in the craniocaudal and dorsal directions to the CTV (prostate and seminal vesicles). The concept included a moderate consecutive dose escalation from 70.2 Gy in 1.8 Gy fractions (n = 206) to 72 Gy in 1.8–2.0 Gy fractions (n = 99 in 1.8 Gy; n = 77 in 2.0 Gy fractions), prescribed to the isocentre. The treatment has not been influenced by this study and the subgroup analysis has not been planned prospectively.

The PTV was required to be enclosed by the 90% isodose relative to the reference point with a margin of 1.5 cm in the anterior/lateral and 1 cm in the craniocaudal and dorsal directions to the CTV (prostate and seminal vesicles). Neoadjuvant and adjuvant hormonal therapy has been added depending on the decision of the referring urologist. This study is based on the principles outlined in the Declaration of Helsinki.

Patients were surveyed using the expanded prostate cancer index composite (EPIC) [8] questionnaire before the beginning of RT. Questionnaires that have been missed in the time between the initial consent and the beginning of RT have not been answered after the start of RT retrospectively (limited time-frame for collection). Long-term questionnaires were sent to the patients 1–2 years, 5–6 years and 9–12 years after the end of RT (minimum changes of five points regarded as clinically significant) with a return envelope. If a questionnaire was not returned within 4 weeks, patients were contacted by telephone and urged to complete it.

The questionnaire comprises 50 items concerning the urinary, bowel, sexual and hormonal domains for function and bothersomeness. The multi-item scale scores were transformed linearly to a 0–100 scale, with higher scores representing better QoL. The Charlson Comorbidity Index (CCI) [9] was calculated as a scale that considers both patient age (starting at 50 years of age, each decade is counted as an extra point) and comorbidities (relevant comorbidities as diabetes or peripheral vascular disease are counted as a point, a localized tumour as two points).

Regular follow-up evaluation was performed by the referring urologist, usually at least every 3 months in the first 2 years, every 6 months up to 5 years and then at least once a year. Follow-up data (laboratory, clinical results) were collected in our department using patient and urologist questionnaires, additionally phone calls and also personal visits at the urologist’s or general practitioner’s practice. Biochemical failure was defined as a rise by 2 ng/ml or more above the nadir (Phoenix definition) and includes patients with an additional hormonal therapy [10].

Statistical analysis was performed using IBM SPSS Statistics 25.0 software (IBM, Armonk, NY, USA). To explore statistical QoL score differences between different subgroups, the Mann–Whitney U-test was used. The Wilcoxon’s matched-pairs test was applied to determine longitudinal changes. Contingency table analysis with the Chi-square test was performed to compare treatment groups with respect to categorical variables. The Kaplan–Meier method was used to assess biochemical recurrence, prostate cancer-specific and overall survival rates. The log-rank test was used to compare survival rates by specific factors. All p-values reported are two-sided, p < 0.05 is considered significant.

## Results

Baseline patient characteristics are presented in Table 1. With the exception of the Gleason score distribution (higher percentage of Gleason score seven and higher in the high dose group), patient characteristics did not change in the subsequent treatment groups. Biochemical tumour control in the 72 Gy subgroup was significantly higher in comparison to the 70.2 Gy subgroup (78% vs. 55% after 10 years; p < 0.01)–including subgroup analyses for intermediate (80% vs. 58%; p < 0.01) and high risk patients (60% vs. 35%; p < 0.01). CCI did not significantly impact prostate cancer-specific survival (84% vs. 78% after 10 years with CCI ≤ 4 vs. CCI > 4; p = 0.07), but overall survival (76% vs. 58% after 10 years with CCI ≤ 4 vs. CCI > 4; p < 0.01). No impact of neoadjuvant hormonal therapy was found on disease-specific or overall survival.

**Table 1:** Baseline patient characteristics

	70.2 Gy/1.8 Gy (n = 206)	72 Gy/1.8-2 Gy (n = 176)
Median follow-up (months)	109 (mean 95)	107 (mean 85)
Median age (range) (years)	71 (45–85)	72 (51–84)
Median planning target volume (range) (cm^3^)	343 (173–627)	336 (169–631)
median initial PSA (range) (ng/ml)	9 (2–104)	8 (2–66)
Gleason score > 6 (%)*	20	48
Neoadjuvant hormonal therapy (%)	35	34
Adjuvant hormonal therapy (%)	11	10
Low^a^/intermediate^b^/high risk^c^ (%)	39 / 31 / 30	35 / 36 / 28
CCI > 4 (%)	70	77

*PSA* prostate-specific antigen, *CCI* Charlson Comorbidity Index

*p < 0.01

^a^No risk factors: PSA < 10 ng/ml, Gleason score < 7, cT-stage < 2b

^b^One risk factor: PSA10-20 ng/ml, Gleason score = 7 or cT-stage = 2b/c

^c^Two risk factors or PSA > 20 ng/ml or Gleason score > 7 or cT-stage > 2b/c

The response rate in the respective intervals considering all treated patients was 78% (n = 297) before RT, 92% (n = 350) 1–2 years, 74% (n = 282) 5–6 years and 52% (n = 199) 9–12 years after the end of RT. The response rate needs to be seen in relation to the 6- year and 12-year overall survival rates of 81% and 58%, respectively. Thus, taking into account only living patients at a specific follow-up, the response rates 5–6 years and 9–12 years after the end of RT were also nearly 90%.

Urinary and bowel bother scores decreased 1 / 3 / 6 points and 7 / 7 / 9 points on average 1 / 5 / 10 years after RT in comparison to baseline scores. The rate of urinary (need of pads in 8% vs. 15% before vs. 10 years after RT; p = 0.01) and bowel (uncontrolled leakage of stool in 5% vs. 12% before vs. 10 years after RT; p < 0.01) incontinence, as well as rectal bleeding (4% vs. 8% before vs. 10 years after RT; p = 0.05) increased. Scores and score changes in the two dose groups are presented in Table 2, specific items in Table 3. In the 72 Gy group, the urinary bother score difference after 5 years was significantly larger in comparison to 70.2 Gy (urinary bother: 6 vs. -1; p = 0.04). A clinically significant decline has only been detected in the 72 Gy group after 5–6 years and 9–12 years. A larger percentage of patients in the 72 Gy group reported big/moderate bother with urinary function overall at all follow-up intervals. Statistically significant longitudinal changes have only been found in the 72 Gy group for urinary function/bother, in contrast to the 70.2 Gy group.

**Table 2:** Mean function/bother scores (quartiles in brackets) before and changes after the irradiation

		Before RT (n = 297)	1–2 years after RT (n = 350)	5–6 years after RT (n = 282)	9–12 years after RT (n = 199)
Urinary function score	70.2 Gy	91 (87/100/100)	− 2 (− 5/0/0)	0 (− 5/0/7)	2 (− 3/0/7)
	72 Gy	92 (88/100/100)	0 (− 5/0/7)	4 (0/0/7)	4 (0/0/7)
Urinary bother score	70.2 Gy	79 (68/86/96)	− 1 (− 10/0/7)	− 1* (− 7/0/11)	3 (− 7/0/14)
	72 Gy	83 (75/89/96)	3 (− 7/0/10)	6* (− 7/4/18)	8 (− 4/4/18)
Bowel function score	70.2 Gy	92 (89/96/100)	3 (− 4/0/7)	2 (− 4/0/7)	5 (− 4/0/9)
	72 Gy	92 (89/96/100)	3 (− 4/0/7)	4 (− 4/0/11)	2 (− 4/0/7)
Bowel bother score	70.2 Gy	93 (93/100/100)	7 (0/0/11)	4 (− 4/0/12)	10 (− 7/0/14)
	72 Gy	94 (93/100/100)	7 (0/0/14)	9 (0/0/14)	8 (0/4/16)
Sexual function score	70.2 Gy	32 (5/33/52)	8 (0/4/18)	17* (0/12/34)	23* (3/25/38)
	72 Gy	29 (4/27/50)	7 (− 3/5/17)	10* (− 3/7/22)	13* (0/12/37)
Sexual bother score	70.2 Gy	60 (25/63/100)	7 (− 9/0/19)	13 (− 6/6/38)	15 (− 6/13/44)
	72 Gy	59 (25/63/100	11 (0/7/31)	16 (0/6/37)	21 (0/25/50)

Positive change = decreasing/worsening quality of life

*RT* radiotherapy

*p < 0.05 comparing the dose groups

**Table 3:** Specific items before RT and at different follow-up times

		Before RT (n = 297)	1–2 years after RT (n = 350)	5–6 years after RT (n = 282)	9–12 years after RT (n = 199)
Need of pads for urinary incontinence (%)	70.2 Gy	8	8	11	12
	72 Gy	8	9	15	19
Big/moderate problem with waking up to urinate (%)	70.2 Gy	31	27	29	29
	72 Gy	22	26	27	29
Big/moderate problem with urinary function overall (%)	70.2 Gy	18	12	16	12
	72 Gy	13	16	22	22
Daily rectal urgency (%)	70.2 Gy	19	15	13	17
	72 Gy	19	15	19	16
Daily stool incontinence (%)	70.2 Gy	5	11	7*	8*
	72 Gy	5	18	19*	17*
> Rare bloody stools (%)	70.2 Gy	2	12	15	5
	72 Gy	6	16	15	11
> Rare painful bowel movements (%)	70.2 Gy	22	30	27	25
	72 Gy	21	28	25	26
Big/moderate problem with bowel function overall (%)	70.2 Gy	7	13	12	12
	72 Gy	4	14	15	15
No ability to have an erection (%)	70.2 Gy	31	47	66	68
	72 Gy	37	47	55	55
Erection not firm enough for intercourse (%)	70.2 Gy	41	78	87	89
	72 Gy	31	76	86	96
Big/moderate problem with sexual function overall (%)	70.2 Gy	32	46	53	43
	72 Gy	37	50	50	46

Numbers correspond to the percentage of patients reporting a symptom or problem

*RT* radiotherapy

*p < 0.05 comparing the dose groups

A higher dose had a statistically significant impact on stool incontinence after 5–6 years and 9–12 years, but also some (statistically not significant) impact on urinary incontinence. Sexual function scores decreased 6, 13 and 18 points in the corresponding intervals (erections sufficient for intercourse in 36% vs. 12% before vs. 10 years after RT; p < 0.01). Sexual function score changes after 5–6 years and 9–12 years were larger in the 70.2 Gy in comparison to the 72 Gy group.

For patients with higher CCI, score changes have not been found significantly different from patients with lower CCI. However, baseline urinary function/bother and baseline sexual function was significantly worse with higher CCI (mean urinary function/bother score of 94/85 vs. 90/80; sexual function score of 41 vs. 27 with CCI ≤ 4 vs. CCI > 4).

Thus, patients who reached the last follow-up 9–12 years after RT had more frequently a lower CCI in comparison to patients who did not reach the last follow-up (CCI > 4 in 64% vs. 81%; p < 0.01), they were younger at the time of RT (median age of 70 vs. 73 years; p < 0.01) and tended to have less aggressive tumours (low/intermediate/high risk in 39%/38%/23% vs. 36%/29%/35%; p = 0.04). Focusing on baseline QoL, a statistically significant difference was found for the urinary function score–94 vs. 89 with vs. without last follow-up; p = 0.02.

## Discussion

Several randomized dose escalation studies have been performed in the last two decades in prostate cancer patients [3]. They have clearly demonstrated improved biochemical tumour control or clinical progression with higher total doses, in line with the results of our study. Increasing the fraction dose from 1.8 Gy to 2.0 Gy and considering an α/β-value of 1.5 for prostate cancer [11], the total dose will be biologically 6% higher, i.e. a total dose of 72 Gy in 2 Gy fractions corresponds to 76.4 Gy in 1.8 Gy fractions. Thus, the dose difference corresponds to 3% with 1.8 Gy fractions (72 Gy/70.2 Gy), but to 9% (76.4 Gy/70.2 Gy) with simultaneous increase from 1.8 Gy to 2.0 Gy fractions. This considerable difference is usually not considered in clinical practice.

As the follow-up of these patients is > 10 years, the dose is rather lower than currently applied in most radiotherapy departments. In contrast to the three-dimensional conformal technique, image-guided and intensity-modulated radiotherapy is regarded as the current standard [12]. Application of these techniques will most probably allow to improve QoL results, as also been shown by rectal spacers that considerably decrease the rectal dose and improve QoL [2, 13, 14].

An International Society of Urological Pathology Consensus Conference of Gleason Grading took place in 2005 [15], with the effect of upgrading many prostate cancers. The percentage of Gleason score 7–10 prostate cancers has increased considerably, with the effect of a larger percentage of higher Gleason scores in the high dose group in our patient population. This effect very likely explains the considerable biochemical control difference between the dose subgroups, as higher Gleason scores have been rated more favourably in earlier years, thus in the patient population treated with lower total doses.

Randomized dose escalation studies have analysed treatment toxicity. The largest effect has been found on gastrointestinal toxicity, with significantly higher grade two or higher rates for higher doses [3]. A correlation of gastrointestinal toxicity with rectal dose–volume parameters is well known [16]. However, quality of life from the patients’ perspective have not been evaluated in these trials.

In a prostate cancer patient population, quality of life changes with longer follow-up cannot be reliably differentiated from changes with ageing [17]. It is known that age and comorbidities have an impact on continence rates and erectile function [17–19]. These are the changes that have also been found in this study, focusing on baseline results for patients with a higher Charlson Comorbidity Index and changes with longer follow-up.

The comparison of different dose levels helps to assess the actual impact of radiotherapy. An effect has been found on urinary bother score changes that have been found to be statically significantly higher in the 72 Gy in comparison to the 70.2 Gy group and have reached the level of clinical significant changes only in the 72 Gy group.

Significant differences of bowel function or bother scores have not been found between different dose levels. However, bowel bother score changes reached the level of clinical significance in both dose groups. A higher dose had a significant impact on stool incontinence rates, additionally a tendency for higher rectal bleeding rates. Smaller margins and more conformal techniques, applying daily image guidance and intensity-modulated techniques, as well as improved rectal protection with prostate-rectum spacers might considerably improve these radiotherapy effects [2, 20].

Several different aspects contribute to sexual function. As presented in Table 3, > 50% of patients still have some erections 9–12 years after the end of radiotherapy (if ability present before treatment). However, this percentage decreased to about 30% if we consider only erections sufficient for intercourse. In contrast to urinary scores, larger sexual function score changes have been found for patients treated with lower total doses. This effect can be well explained by higher recurrence rates with the need of antiandrogen treatment in the low dose group.

## Conclusion

The dose escalation was found to be clinically relevant for biochemical tumour control and quality of life. This is the consequence of a simultaneous increase of the dose per fraction from 1.8 Gy to 2 Gy. Apart from an increasing rate of erectile dysfunction, urinary and bowel incontinence rates increased with increasing follow-up period. A higher dose was found to be associated with increased urinary problems and larger stool incontinence rates. Age and comorbidities were found to be relevant for baseline scores, but not for score changes.

## Data Availability

Data are available at the Department of Radiation Oncology, RWTH Aachen University. Patients did not approve to a public data deposition.

## References

[1] Pinkawa M, Piroth MD, Holy R, Djukic V, Klotz J, Krenkel B (2011). Combination of dose escalation with technological advances (intensity-modulated and image-guided radiotherapy) is not associated with increased morbidity for patients with prostate cancer. Strahlentherapie und Onkologie.

[2] Ghadjar P, Fiorino C, MunckAfRosenschold P, Pinkawa M, Zilli T,  vanderHeide UA (2019). ESTRO ACROP consensus guideline on the use of image guided radiation therapy for localized prostate cancer. Radiotherapy and Oncology.

[3] Viani GA, Stefano EJ, Afonso SL (2009). Higher-than-conventional radiation doses in localized prostate cancer treatment: A meta-analysis of randomized, controlled trials. International Journal of Radiation Oncology Biology Physics.

[4] Kuban DA, Levy LB, Cheung MR, Lee AK, Choi S, Frank S (2011). Long-term failure patterns and survival in a randomized dose-escalation trial for prostate cancer Who dies of disease?. International Journal of Radiation Oncology Biology Physics.

[5] Ozyigit G, Onal C, Igdem S, Alicikus ZA, Iribas A, Akin M (2019). Treatment outcomes of prostate cancer patients with Gleason score 8–10 treated with definitive radiotherapy : TROD 09–001 multi-institutional study. Strahlentherapie und Onkologie.

[6] Kikkawa K, Iba A, Kohjimoto Y, Noda Y, Sonomura T, Hara I (2018). Impact of age on quality of life in patients with localized prostate cancer treated with high-dose rate brachytherapy combined with external beam radiotherapy. International Journal of Urology.

[7] Schlenter M, Berneking V, Krenkel B, Mottaghy FM, Vogeli TA, Eble MJ (2018). Intensity-modulated radiotherapy of prostate cancer with simultaneous integrated boost after molecular imaging with 18F-choline-PET/CT : Clinical results and quality of life. Strahlentherapie und Onkologie.

[8] Wei JT, Dunn RL, Litwin MS, Sandler HM, Sanda MG (2000). Development and validation of the expanded prostate cancer index composite (EPIC) for comprehensive assessment of health-related quality of life in men with prostate cancer. Urology.

[9] Charlson ME, Pompei P, Ales KL, MacKenzie CR (1987). A new method of classifying prognostic comorbidity in longitudinal studies: development and validation. J Chronic Dis.

[10] Roach M, Hanks G, Thames H, Schellhammer P, Shipley WU, Sokol GH (2006). Defining biochemical failure following radiotherapy with or without hormonal therapy in men with clinically localized prostate cancer: Recommendations of the RTOG-ASTRO Phoenix Consensus Conference. International Journal of Radiation Oncology Biology Physics.

[11] Fowler JF, Toma-Dasu I, Dasu A (2013). Is the alpha/beta ratio for prostate tumours really low and does it vary with the level of risk at diagnosis?. Anticancer Research.

[12] Pinkawa M, Schoth F, Bohmer D, Hatiboglu G, Sharabi A, Song D (2013). Current standards and future directions for prostate cancer radiation therapy. Expert Review of Anticancer Therapy.

[13] Chao M, Ow D, Ho H, Chan Y, Joon DL, Spencer S (2019). Improving rectal dosimetry for patients with intermediate and high-risk prostate cancer undergoing combined high-dose-rate brachytherapy and external beam radiotherapy with hydrogel space. J Contemp Brachytherapy.

[14] Miller LE, Efstathiou JA, Bhattacharyya SK, Payne HA, Woodward E, Pinkawa M (2020). Association of the placement of a perirectal hydrogel spacer with the clinical outcomes of men receiving radiotherapy for prostate cancer: A systematic review and meta-analysis. JAMA Netw Open.

[15] Epstein JI, Allsbrook WC, Amin MB, Egevad LL (2005). The International Society of Urological Pathology (ISUP) consensus conference on gleason grading of prostatic carcinoma. American Journal of Surgical Pathology.

[16] Paleny R, Bremer M, Walacides D, Mainwaring S, Weber K, Henkenberens C (2019). Comparison of relative and absolute rectal dose-volume parameters and clinical correlation with acute and late radiation proctitis in prostate cancer patients. Strahlentherapie und Onkologie.

[17] Resnick MJ, Koyama T, Fan KH, Albertsen PC, Goodman M, Hamilton AS (2013). Long-term functional outcomes after treatment for localized prostate cancer. New England Journal of Medicine.

[18] Pinkawa M, Fischedick K, Gagel B, Piroth MD, Asadpour B, Klotz J (2009). Impact of age and comorbidities on health-related quality of life for patients with prostate cancer: evaluation before a curative treatment. BMC Cancer.

[19] Leufgens F, Berneking V, Vogeli TA, Kirschner-Hermanns R, Eble MJ, Pinkawa M (2019). Quality of life changes >10 years after postoperative radiation therapy after radical prostatectomy for prostate cancer. International Journal of Radiation Oncology Biology Physics.

[20] Pinkawa M, Berneking V, Schlenter M, Krenkel B, Eble MJ (2017). Quality of life after radiation therapy for prostate cancer with a hydrogel spacer: 5-year results. International Journal of Radiation Oncology Biology Physics.

